# Reconstruction of ancestral RNA sequences under multiple structural constraints

**DOI:** 10.1186/s12864-016-3105-4

**Published:** 2016-11-11

**Authors:** Olivier Tremblay-Savard, Vladimir Reinharz, Jérôme Waldispühl

**Affiliations:** 1 School of Computer Science, McGill University, Montreal, H3A 0E9 Canada; 2 Department of Computer Science, University of Manitoba, Winnipeg, R3T 2N2 Canada

**Keywords:** RNA, Secondary structure, Ancestor reconstruction, Evolution, Phylogeny, Algorithm

## Abstract

**Background:**

Secondary structures form the scaffold of multiple sequence alignment of non-coding RNA (ncRNA) families. An accurate reconstruction of ancestral ncRNAs must use this structural signal. However, the inference of ancestors of a single ncRNA family with a single consensus structure may bias the results towards sequences with high affinity to this structure, which are far from the true ancestors.

**Methods:**

In this paper, we introduce achARNement, a maximum parsimony approach that, given two alignments of homologous ncRNA families with consensus secondary structures and a phylogenetic tree, *simultaneously* calculates ancestral RNA sequences for these two families.

**Results:**

We test our methodology on simulated data sets, and show that achARNement outperforms classical maximum parsimony approaches in terms of accuracy, but also reduces by several orders of magnitude the number of candidate sequences. To conclude this study, we apply our algorithms on the Glm clan and the FinP-traJ clan from the Rfam database.

**Conclusions:**

Our results show that our methods reconstruct small sets of high-quality candidate ancestors with better agreement to the two target structures than with classical approaches. Our program is freely available at: http://csb.cs.mcgill.ca/acharnement.

**Electronic supplementary material:**

The online version of this article (doi:10.1186/s12864-016-3105-4) contains supplementary material, which is available to authorized users.

## Background

With the development of sequencing technologies emerged the need to elucidate the relationship between sequences from various organisms. The reconstruction of ancestral sequences, which aims to reveal the chain of events that led to the diversity of sequences observed today, became naturally one of the core challenges in this field of research. Since the first attempts to rigorously solve this problem [1], the methods and quality of the data have considerably improved, to the point where the reconstruction of ancient genomes is now feasible [2–4].

For a long time, most of the attention has been given to the reconstruction of ancient protein and DNA sequences, while RNA molecules remained relatively overlooked. Nonetheless, in the last 20 years, the discovery of the breadth of catalytic and regulatory functions carried by RNA molecules revived our interest for the RNA world hypothesis [5], and resulted in increasing efforts toward a better understanding of the intricate nature of mutational patterns in RNAs [6–11].

The reconstruction of non-coding RNA (ncRNA) sequences is particularly challenging. Indeed, ncRNA functions are typically carried out by specific molecular structures, and consequently sequences are generally less conserved than structures [12]. This implies that dedicated frameworks must be developed to capture this structural information.

RNA folding is hierarchical. Secondary structures form rapidly and act as a scaffold for the slower formation of tertiary structures [13]. It follows that the stability of secondary structures provides us a relatively accurate signature of the molecular function [14], and thus can be used to guide the reconstruction of ancestral ncRNA sequences.

To date, the most promising approach to infer ncRNA ancestors has been proposed in 2009 by D. Bradley and I. Holmes, who introduced an algorithm to calculate ancestral RNA secondary structures from an alignment [15], and use these structures to infer ancestral sequences using a maximum-likelihood approach on stochastic grammars [16]. Still, the time complexity of inferring ancestral structures can be prohibitive, and the specificity of the functional structure may not accommodate sufficiently large variations of this (secondary) structure to take advantage of this model.

**Fig. 1:**
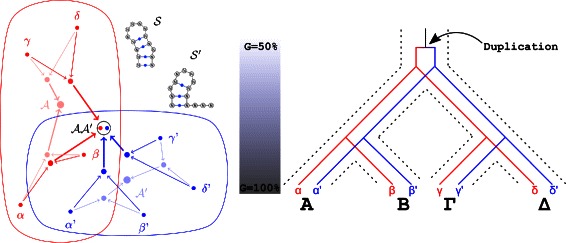
Our approach. *Left:* The red and blue areas represent regions of the sequence landscape of sequences with “good” affinity (i.e. sufficient to carry the associated function) to the target structures $\mathcal {S}$ (*red*) and $\mathcal {S}'$ (*blue*). Here, *α* and *α* ^′^ are paralogous sequences, as well as *β* and *β* ^′^, *γ* and *γ* ^′^ and *δ* and *δ* ^′^. Using classical reconstruction approaches, $\mathcal {A}$ would be the inferred ancestor of the orthologous sequences *α*, *β*, *γ* and *δ*, and $\mathcal {A}'$ would be the inferred ancestor of the orthologous sequences *α* ^′^, *β* ^′^, *γ* ^′^ and *δ* ^′^. Shaded trees represent the classical ancestral reconstructions performed separately, while the main tree rooted at $\mathcal {A}\mathcal {A}'$ represents the *simultaneous* ancestral reconstruction approach introduced in this contribution. The rationale of this work is that ancestors inferred from a single family and structure may have a tendency to be located in the core of the affinity regions, and might end up with ancestral sequences that would be hard to reconcile. By contrast, a simultaneous reconstruction of orthologous families ensures the coherency of the process and a better inference of the ancestors (which are not necessarily located in the core of the affinity regions). *Right*: An example of a species tree *T* (*dashed lines*) of four species *A*, *B*, *Γ* and *Δ* corresponding to the neutral networks shown on *the left*. A duplication event is shown at the root, creating the two ncRNA families (represented by colored lines). Each node of the species tree contains a copy of each ncRNA family (*one red, one blue*). At the leaves of the species tree *T*, we find the two extant ncRNAs for which we have the sequence and the structure information. The linear gradient G is also shown: it represents the weight that is given to each structure when calculating the costs (G for one structure and 100 %-G for the other)

Covariation models are powerful frameworks to model families of structured RNA sequences [17–19], allowing us to capture dependencies between distant sites. Nevertheless, we argue that the reconstruction of ancestral RNA sequences of a single ncRNA family with a single secondary structure using a covariation model can be hazardous. Indeed, current sequences are most likely uniformly distributed on the entire neutral network of the functional structure [20] (i.e. regions of the sequence landscape with a good affinity to the functional structure), and a strategy aiming to accommodate constraints within a single family will have a tendency to produce ancestors near the core of this network. This bias may result in ancestral sequences potentially far from the first ancestor who acquired the function (*i.e.* the structure). In other words, this first ancestor is likely to be a worse fit to the functional structure than sequences at the core of the neutral network. By contrast, in this paper we adopt a radically different approach. We propose here to solve this problem simultaneously for two ncRNA families that share a common ancestor (See Fig. 1). This strategy enables us to make a better estimation of the location of the duplication event at the origin of the two families in the sequence landscape, hence to make a more accurate inference of the ancestors of each family.

Our approach is as follows. Given two alignments of homologous ncRNA families with consensus secondary structures and a phylogenetic tree, we design a maximum parsimony algorithm to *simultaneously* compute ancestral RNA sequences for both families. We test this methodology on simulated data sets, and compare the results to classical (structure-free) maximum parsimony approaches [21, 22], as well as to a customized maximum parsimony algorithm integrating the constraints of a single structure. Finally, we apply our techniques to the reconstruction of ancestral sequences of two Clans (Glm [23] and FinP-traJ [24]) from the Rfam database [25]. Clans are RNA families that “share a common ancestor but are too divergent to be reasonably aligned” [26], and thus illustrate well the signal we aim to capture.

Our results on simulated data sets show that our strategy improves the accuracy of the reconstruction. On real data sets, our approach compares favorably to PAML, a state-of-the-art maximum likelihood method that considers one structure at a time, and customized versions of the Fitch and Sankoff algorithms. In particular, our data shows that our solutions have a better agreement to the two target structures than the sequences inferred with previous methods. Importantly, we achieve all these results with a significantly smaller set of candidate ancestors, which improves the interpretability of our data.

Our algorithms have been implemented in a software named achARNement and are freely available at http://csb.cs.mcgill.ca/acharnement.

## Methods

### Input data

For the algorithms presented in this paper, we assume that we have two non-coding RNA families that have been identified as a clan [26]. For each of the two ncRNA families, we have the consensus 2D structure it folds into. We also have a set of species that each possess one copy of both ncRNAs (one of each family), and a species tree *T* that represents the speciation history of the organisms considered. We have the sequences of the two ncRNAs for each of the studied species. Figure 1 illustrates an example of a species tree.

### Problem statement

Given the input data described in the previous subsection, the problem is to infer a most parsimonious set of ancestral sequences for each of the two ncRNAs at each ancestral node of the input species tree. Although this is a very classical problem in comparative genomics, our goal is to achieve that using a new evolutionary model that simultaneously considers sequence and 2D structure information, as described previously.

### Evolutionary model

Our evolutionary model assumes that the two ncRNA families are the result of an ancient duplication of an ancestral ncRNA that was able to fold into two different structures. Following the duplication, a subfunctionalization process took place: a series of neutral mutations occurred and gave rise to both extant families that can only fold into one specific structure (see Fig. 1). Here, we assume that the ancestor of all studied species already possessed both ncRNAs, but that the duplication event occurred not too long before that (near the root of the species tree *T* representing the studied organisms). Only point mutations are allowed in our evolutionary model (no indels).

As mentioned earlier, ncRNA sequences are more constrained by their structure than their sequence during evolution. Since we have only access to the 2D structure of the two extant families (and not the ancestral 2D structure), our model considers both of these structures during the inference process. Near the root of the species tree, our model suggests that the sequences were still likely to be able to fold into both structures. However, as time passes, each ncRNA starts to specialize into only one structure and loses affinity to the other. We represent that gradual transition into our model using a gradient *G* which varies from 50 *%* (near the root) to 100 *%* (near the leaves). This gradient is going to be used in our algorithm to calculate the “weight” that each of the structures must have in the global score of the inferred ancestral sequences.

We developed two novel algorithms, implemented in a package called achARNement and freely available at http://csb.cs.mcgill.ca/acharnement.

### Algorithms

**Fig. 2:**
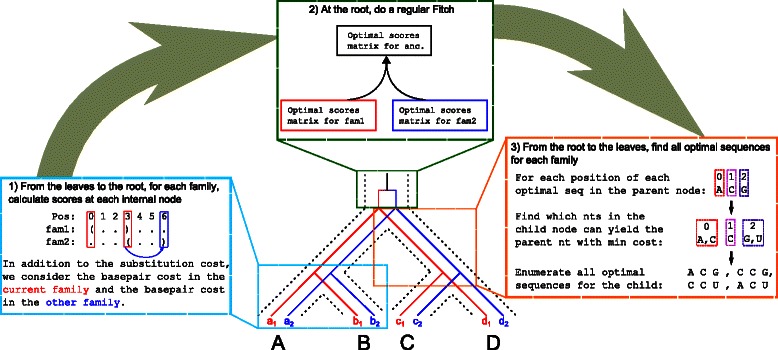
Graphical representation of the algorithm CalculateScores-2structs. In this example, we have four species (*A, B, C* and *D*) and for each species, we have two extant RNAs (for family 1, in *red*, and family 2, in *blue*). The three major steps of the algorithm are presented. 1) The *bottom-up step*, where minimum scores are calculated at every node of the tree for each family. The scores take into account the substitutions, but also the basepair cost for the current family, and for the other family. 2) The *middle step*. Here we link the minimum score matrices for families 1 and 2 by doing a simple Fitch on the two matrices. This allows us to reconstruct the original ancestral sequences (before the duplication), taking into account both families. 3) The *top-down step*, where we start from the root and select the nucleotides of minimum cost at every position and construct the optimal sequences

We propose a new tool, achARNement, composed of two exact algorithms (CalculateScores-1struct and CalculateScores-2structs) based on the Fitch [21] and Sankoff [22] parsimony methods for the inference of ancestral sequences in a phylogeny (note that we are focusing on the inference of ancestral sequences, and not only on the calculation of parsimony scores). Our algorithms use a three-step approach (see Fig. 2): (i) a bottom-up step in which minimal costs for every possible nucleotide at every site are calculated, (ii) a middle step where we link the minimal cost matrices for both families at the root of the phylogeny, and (iii) a top-down step that enumerates all the optimal sequences based on the calculated costs. Our algorithms have the same running-time complexity than the Sankoff algorithm (*O*(*N* *k*), where *N* is the number of nodes and *k* is the sequence length); the only difference being a constant number of additional calculations that depends on the basepairs in the two structures.

For the substitutions, we use a cost matrix that has a different weight for transitions and transversions, since transversions normally occur less frequently than transitions (see Table 1). In addition to the substitution cost, we also consider a basepair cost, as shown in Table 2. The basepair cost is 0 for the G-C basepairs and 0.001 for the A-U basepairs, that are not as strong as G-C basepairs. Compared to an A-U basepair, a G-U pair costs twice as much, while all the others are penalized three times as much. We have also experimented with a more complex scoring system for the basepairs, one that reflects the geometry and isostericity of the basepairs. We performed tests using the IsoDiscrepancy Index (IDI) table, as described in [27]. However, since this table represents a transition from the initial basepair to a mutated basepair, more calculations were required by our CalculateScores-2structs algorithm. The results obtained with the IDI table and our simpler table (Table 2) were very similar (results not shown), but at the cost of a 4-fold increase in computation time. Consequently, we decided to use the simpler table.

**Table 1:** Nucleotide substitution matrix

	A	C	G	U
A	0	2	1	2
C	2	0	2	1
G	1	2	0	2
U	2	1	2	0

Each cell *C*_*i*,*j*_ represents the cost of mutating the nucleotide i into j

**Table 2:** Basepair cost matrix

	A	C	G	U
A	0.003	0.003	0.003	0.001
C	0.003	0.003	0	0.003
G	0.003	0	0.003	0.002
U	0.001	0.003	0.002	0.003

Each cell *C*_*i*,*j*_ represents the cost of having the basepair i-j

The difference between the two algorithms we propose (CalculateScores-1struct and Calculate Scores-2structs) resides in the first step (bottom-up), where we calculate the minimal costs for every possible nucleotide at every site. Let *f* be one of the two families and $\bar {f}$ be the other one. When calculating the costs for family *f* at the internal node *a*, algorithm CalculateScores-1struct considers only the structure associated with family *f*. On the other hand, algorithm CalculateScores-2structs considers both structures, but with a weight *G* that varies along the depth of the tree. For example, at the level of the leaves, for the family *f*, we consider 100 % of the structure *f* and 0 % of the structure $\bar {f}$. At the level of the root, we consider 50 % of the structure of family *f* and 50 % of the structure of family $\bar {f}$. We use a linear gradient to set the values of *G* on the different depths of the tree (from 50 to 100 %).

For space reasons, the full description of the algorithms was placed in the Additional file 1. In the following paragraphs, an overview of the algorithms will be presented.

#### Bottom-up step

The first step of the algorithms consists of doing a post-order traversal of the species tree (as shown in Algorithm 1, Additional file 1), to calculate the most parsimonious costs for each possible nucleotide at every site.

In the following paragraphs, we explain the differences between CalculateScores-1struct and CalculateScores-2structs in the calculation of those costs.

##### CalculateScores-1struct:

Let *a*_*i*_ be the nucleotide at position *i* in the parent (ancestral) node, $\bar {a_{i}}$ be the nucleotide that is paired with *a*_*i*_ in the current structure, *l*_*i*_ (resp. *r*_*i*_) be the nucleotide at position *i* in the left (resp. right) child, and $\bar {l_{i}}$ (resp. $\bar {r_{i}}$) be the nucleotide that is paired with *l*_*i*_ (resp. $\bar {r_{i}}$) in the left (resp. right) child in the current structure.

In the case that the position *i* is part of a basepair in the current structure, the cost of having a specific dinucleotide $a_{i},\bar {a_{i}}$ is equal to:

1 $$ \begin{aligned} &\min_{l_{i} \text{and} \bar{l_{i}} \in \{A,C,G,U\}} \!\{c(l_{i})\! + s(l_{i},a_{i}) +\! c(\bar{l_{i}}) \,+\, s(\bar{l_{i}},\bar{a_{i}}) \,+\, bpc(a_{i},\bar{a_{i}})\} \\ &+\min_{r_{i} \text{and} \bar{r_{i}} \in \{A,C,G,U\}} \!\{c(r_{i}) \,+\, s(r_{i},a_{i}) \,+\, c(\bar{r_{i}})\! +\! s(\bar{r_{i}},\bar{a_{i}}) \,+\, bpc(a_{i},\bar{a_{i}})\} \end{aligned}  $$

where *c*(*x*) is the previously calculated optimal cost of having the nucleotide *x*, *s*(*x*,*y*) is the cost of substituting nucleotide *x* for *y* and *b* *p* *c*(*x*,*y*) is the cost of having the basepair (*x*,*y*). In the other case where *i* is not part of a basepair, we simply calculate the substitution costs.

##### CalculateScores-2structs:

**Fig. 3:**
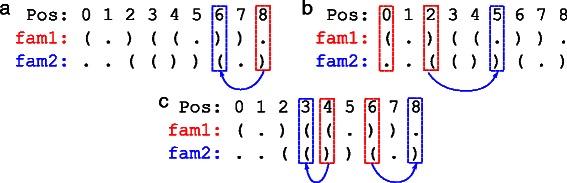
Three examples of the positions that need to be considered when using information from both structures. Note that in those examples, we consider that we are working on the sequence of family 1, and fam1 and fam2 represent the 2D structures of family 1 and 2 respectively. **a** The easier case when the position (8 here) is not paired in fam1, and we only have to consider the position paired with it in fam2. **b** The case where only one of the two paired positions of fam1 is paired in fam2. **c** The case where both paired positions of fam1 are paired in fam2

As mentioned earlier, CalculateScores-2structs takes into account both structures, using a weight *G*. Calculating the costs on the left and right branches is a little bit different depending on if we are dealing with a paired position or an unpaired one. The general idea is that for each position *i*, we are going to measure the cost of the basepair formed with position $\bar {i}$ (if it exists) in the structure of the current family, and we are also going to consider the positions paired with both *i* and $\bar {i}$ in the other structure. Note that each position can be paired to two different positions in the two structures; we will focus on that case here, because if the basepairs are the same in both structures, then we do not need the gradient *G* and simply consider 100 % of the basepair cost. Figure 3 shows three examples.

The simpler case is when the position *i* is unpaired. Then, only the position paired with *i* in the other structure needs to be considered, if it is paired (see Fig. 3 a). Since that position (e.g. position #6 in Fig. 3 a) is not necessarily fixed, we consider an average basepair cost over all possible nucleotides at that position.

The more complex case is when position *i* is paired.

In this case, we also have to check for the position paired with $\bar {i}$ in the other structure (see Fig. 3 b and c).

More precisely, using the same definitions as above for CalculateScores-1struct, and considering that position *i* is paired in both structures (and position $\bar {i}$ too), the cost of having a specific dinucleotide $a_{i},\bar {a_{i}}$ is equal to:

2 $$ \begin{aligned} {}Eq. (1)&\left[\text{weighting}\, bpc(a_i, \bar{a_i})\, \text{by}\, G \right] \,+\, (1\,-\,G)\\ &\quad\!*\! \!\left(\sum\limits_{nc \in \{A,C,G,U\}} bpc(a_{i},nc)/4 + \sum\limits_{nc \in \{A,C,G,U\}} bpc(\bar{a_{i}},nc)/4\!\right) \end{aligned}  $$

Note that it is possible to get cycles of “interdependent” positions when considering both structures. As you can observe in Fig. 3 c, positions 4 and 6 are paired together in fam1. In fam2, position 4 is paired with 3, which is paired with position 7 in fam1. Finally, position 6 is paired with position 8 in fam2. Thus all of those positions are “interdependent”. To simplify the algorithm, instead of considering the complete cycles, we chose to stop at one “level”, that is looking only at one paired position in the other structure for each position in the first structure.

Once the costs are calculated for every site at every node of the species tree, we can simply do the middle and top-down steps.

#### Middle and top-down steps

The top-down step is the part where we start from the root of the tree, we select the nucleotides of minimum cost at every position and construct the optimal sequences. Once all the optimal sequences are enumerated at an internal node of the tree, we go down in the tree and enumerate the optimal sequences that gave rise to them in the child nodes and so on. Algorithm 6 (Additional file 1) describes this process. Note that before starting to select the nucleotides at the root, we do a simple Fitch (algorithm not shown here) on both cost matrices of family 1 and 2. This middle step is necessary to make the link between the two families, *i.e* reconnect both matrices of optimal scores, and reconstruct the original ancestral sequences (before the duplication).

#### Generalizing to more than two families

This problem can easily be generalized to *F*>2 structure families, as long as we maintain the same assumption that all ancestors represented in the tree possess one copy of each *F* number of ncRNAs. The only part of the algorithm that would change is the bottom-up step: it would be similar to CalculateScores-2structs, except that we would be considering the basepairs in all the *F* structures instead of just two. The gradient *G* would also be different: it would range from $\frac {100}{F}\,\%$ (near the root, where the same weight is given to all structures) to 100 *%* (near the leaves).

## Results and Discussion

### Simulated data generation

To evaluate our method, we generated in silico twenty different phylogenetic trees for three different pairs of secondary structures as follows. First, three secondary structures of size 100 were randomly designed such that the two first have a similar shape, and the last, a different one. Those structures are the following


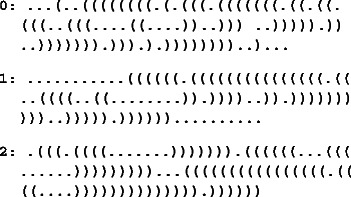


The base pair distances evaluated with RNAdistance [28] between the structures 0 and 1, 0 and 2, and 1 and 2 are respectively 40, 96 and 86.

For every pair of secondary structures, a set of twenty bi-stable sequences was generated with Frnakenstein [29], such that the best scoring sequence of each run was kept. For each pair of structures (*s*_1_,*s*_2_), and each sequence *z* designed on these structures, a complete binary tree *T* of depth 6 was populated. The root *r* of *T* is initialized with (*r* ^1^,*r* ^2^)≡(*z*,*z*). Each internal node *n* of *T* is composed of a pair of sequences, (*n* ^1^,*n* ^2^), such that the sequence *n* ^1^ is associated with the structure *s*_1_ and *n* ^2^ with the structure *s*_2_.

The sequences for each internal node are generated in a top-down fashion. Given a node *w*, its sequences (*w* ^1^,*w* ^2^), its two child nodes *c* ^1^,*c* ^2^, a mutation probability *α*, and a substitution matrix *β*. From (*w* ^1^,*s*_1_) (resp (*w* ^2^,*s*_2_)), a set *W* of a thousand mutants of *w* ^1^ is generated as follows.

Each sequence *w*_*i*_ in *W* is created by applying a probability of mutation *α* to each position in the original sequence *w* ^1^.

Each nucleotide *x* can be substituted to {A,C,G,U}∖{*x*} following the distribution *β*(*x*). We used for *β*: $\mathbb P\left (\mathrm {A}\leftrightarrow \mathrm {G}\right) = \mathbb P\left (\mathrm {C}\leftrightarrow \mathrm {U}\right) = 50\, \%$, all others are set to 25 *%*. We used those probabilities for the mutational events based on the observation [30] that transitions are more frequent than transversions.

We define a free energy *E*(*w*_*i*_,*s*) as the base pair distance between the minimal free energy structure of *w*_*i*_ and *s*, i.e. *Δ*(MFE(*w*_*i*_),*s*). The MFE and base pair distance are computed with RNAfold and RNAdistance [28].

A Boltzmann distribution is induced such that the weight of any sequence *w*_*i*_ is

$$\mathcal B(w_{i}, s) = e^{\frac{-E(w_{i}, s)}{RT}} $$

where *R* is the Boltzmann constant and *T* the temperature in Kelvin. The partition function $\mathcal {Z}^{s}_{W}$ is obtained by summing the weights of all sequences *w*_*i*_∈*W* and we defined the Boltzmann probability of each sequence $\mathbb {P}^{s}_{W}(w_{i})$ such that

$$ \mathcal{Z}_{W}^{s} = \sum\limits_{w_{i}\in W} \mathcal{B}(w_{i}, s) \qquad \text{and} \qquad \mathbb{P}_{W}^{s}(w_{i})=\frac{\mathcal{B}(w_{i}, s)}{\mathcal{Z}^{s}}. $$

We sample two sequences from this distribution to populate ${c^{1}_{1}}$ and ${c^{2}_{1}}$ (resp. ${c^{1}_{2}}$ and ${c^{2}_{2}}$). We re-apply recursively. The generator was implemented in python and is bundled with our achARNement package.

### Evaluation on simulated data

We first evaluated achARNement using simulated data, as described in Sec. Simulated data generation. The mutational rates of bacterias (bacterial genomes are studied in Sec. Evaluation on biological data) are known to vary greatly between species and it is difficult to find indisputable reference points to evaluate them [31]. We thus approximate many generations in each step (*i.e* level of the tree) by using as the mutation rate *α* three values: {1 *%*,5 *%*,10 *%*}. This enables us to obtain diverse enough sequences at the leaves of a complete binary tree of depth 6.

**Fig. 4:**
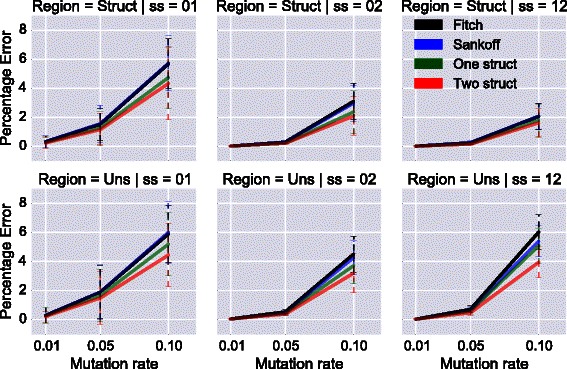
The average error percentage of all optimal sequences for both families in a tree. Each column represents a pair of secondary structures. The *first row* is for positions in structured regions, and the *second row* for unstructured regions. For three mutation rates: 1 *%*, 5 *%* and 10 *%*

For every pair of structures and mutation rate, twenty trees were generated. In Fig. 4, we show the average error percentage over all optimal sequences inferred for both families in all nodes of the trees. We divided the results by structural features; the first row is the average error percentage for positions involved in an interaction, while the second row is for unstructured positions. Each column represents a different pair of secondary structures, annotated 01, 02 and 12 following the notation defined in Sec. Simulated data generation. For each sequence of a family *fam*, we consider a position to be in a structured region, if the structure of *fam* has a base pair at that position.

A first observation is that CalculateScores- 2structs always performs the best, followed by CalculateScores-1struct, and the Fitch and Sankoff algorithms whose performances are equivalent. In all cases, achARNement methods always perform better, even in unstructured regions.

For CalculateScores-1struct, although the other structure is ignored during the parallel ancestral reconstructions, some constraints from the other structure are implicitly taken into account during the middle step when solutions from both families are merged. The higher quality in unstructured regions when using CalculateScores-2structs was expected because we always consider structures from both families, and one unstructured position in one family can be structured in the other. Finally, although the two structures 0 and 1 are much closer to each other than to 2, the basepair distance does not seem to affect the quality of the results.

**Fig. 5:**
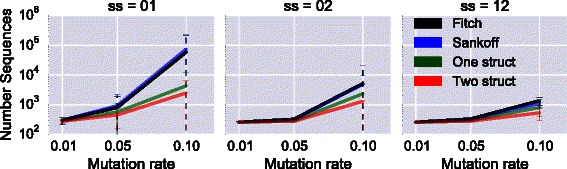
Average number of optimal sequences in the **tree**, y-axis logscale. Each column represents a different pair of secondary structures. For three mutation rates: 1 *%*, 5 *%* and 10 *%*

**Fig. 6:**
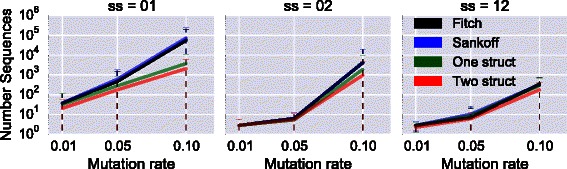
Average number of optimal sequences in the **root**, y-axis logscale. Each column represents a different pair of secondary structures. For three mutation rates: 1 *%*, 5 *%* and 10 *%*

We then examine the number of optimal solutions, for each pair of secondary structures and mutation rate *α*. As can be observed in Figs. 5 and 6, the average number of optimal sequences inferred both in the whole tree and for the root only is always smaller for algorithms CalculateScores-1struct and CalculateScores-2structs, compared to Fitch and Sankoff. In the case of the pair of structures 01, the average number of optimal sequences is even several orders of magnitude lower for our two algorithms. An important observation is that, in every case, all sequences at the root reconstructed by CalculateScores-2structs are a **subset** of the optimal sequences obtained with the classical Sankoff algorithm (*i.e.* CalculateScores-Sankoff). This shows that the additional structural constraints defined in our method help to reduce the initial solution space produced by traditional approaches.

Running times for the four methods are shown in Additional file 1 Sec. Running times.

### Evaluation on biological data

We analyzed the Glm and FinP-traJ clans from the Rfam database. A clan contains two RNA families, that are homologous but functionally and structurally distinct [26]. These clans, with their two functional families with distinct consensus structures, constitute good candidates to test our algorithms.

#### Glm clan

The Glm clan contains two bacterial small non-coding RNAs, GlmY and GlmZ, that are homologous but functionally distinct. They act in a hierarchical manner to activate the translation of the glmS mRNA [23]. We selected 74 bacterial genomes for which Rfam alignments were available for both families (see the complete list in Additional file 1 Sec. Biological Data). The phylogeny of the 74 studied bacterial strains was taken from the Pathosystems Ressource Integration Center (PATRIC) [32], and Rfam seed alignements of both families were aligned together with CARNA [33]. The sequences in the full Rfam alignments were then re-aligned to the alignment obtained with CARNA simply by mapping their corresponding positions. The sequences and structures were subsequently trimmed to remove the gapped columns, and if only one side of an interaction was removed the other position was marked as unstructured.

We used the basic Fitch and Sankoff methods, and our algorithms CalculateScores-1struct and CalculateScores-2structs to infer the ancestral sequences at the root of the species tree. Both Fitch and Sankoff inferred the same set of 786 432 sequences at the root of the species tree, whereas CalculateScores-1struct inferred 393 216 and CalculateScores-2structs 196 608. The ancestral sequences reconstructed by our methods are subsets of the ones produced by Fitch and Sankoff: CalculateScores-1struct cut the solution space in half and CalculateScores- 2structs by another half. Running times were of 19 seconds for both Fitch and Sankoff, and 14 seconds for both CalculateScores-1struct and CalculateScores-2structs. The lower running times for achARNement methods could be explained by the smaller numbers of ancestral sequences inferred.

We look at two different measures to evaluate the quality of the ancestral sequences. First, we simply look at the percentage of all structured positions, for each family, that can actually form canonical basepairs in the ancestral sequences. The goal is to see if the reconstructed sequences can form the required basepairs in both structures. Second, we compute the harmonic mean (H-mean) between the frequencies in the ensemble of structures for each structure family (representing GlmY and GlmZ). In a statistical physics framework, an RNA sequence can adopt all structures and its frequency represents the fraction of time that the sequence adopts a particular structure. The harmonic mean is defined as

$$2 \cdot \frac{FreqS1 \times FreqS2}{FreqS1 + FreqS2} $$

and is maximized when both frequencies are at 0.5, given that the structures are different. Thus the H-mean will be equal to 0.5 if the two structures are different and share the complete structure space. Another important feature is the energy of a sequence in a particular configuration. Although that sequence could have other more favorable structures, it gives another idea of the stability of a particular configuration.

In order to calculate this mean for a sequence, we compute the free energy of the sequence when folded in the 2 different structures and their frequencies using RNAfold, and the non-canonical base pairs are ignored for these computations.

To compare the different ensembles of solutions, we sampled 200 000 distinct sequences from each of them. We present in the first 6 lines of Table 3 the maximum and average values of the percentage of canonical basepairs and H-mean for: sequences inferred by Sankoff (or Fitch) only, those inferred by CalculateScores-1struct but not CalculateScores-2structs, and those inferred by CalculateScores-2structs only. We also present the values of energy and frequencies in regards to the secondary structure of each family. The standard deviations are shown in the Additional file 1: Tab. 7.

**Table 3:** Maximum and average results for the Glm Clan

	%Z	%Y	H-mean	EnSZ	FreqSZ	EnSY	FreqSY
Sankoff	100	100	1.40e-03	-18.7	1.40e-03	-18.7	1.40e-03
average	99.1	93.0	6.60e-06	-15.5	4.09e-06	-17.8	1.55e-04
1struct	100	100	8.76e-03	-19.3	8.76e-03	-19.3	8.76e-03
average	99.1	93.0	9.02e-05	-16.1	5.54e-05	-17.4	5.68e-04
2struct	100	100	2.45e-03	-19.3	1.27e-03	-21.3	3.26e-02
average	99.1	94.5	7.45e-06	-16.1	3.78e-06	-19.4	9.27e-04
PAML GlmZ	92.9	90.6	1.53e-05	-18.1	7.66e-06	-22.6	1.14e-02
PAML GlmY	92.9	90.6	1.98e-07	-17.5	9.90e-08	-22.5	3.30e-04

The %Z (resp. %Y) column shows the percentage of all structured positions in the GlmZ (resp. GlmY) family for which the ancestral sequences can form canonical basepairs. The H-mean column represents the harmonic mean. The EnSZ column (resp. EnSY) shows the energy of the sequence when folded in the secondary structure of the family GlmZ (resp. GlmY). The FreqSZ column (resp. FreqSY) shows the frequency in the ensemble of the secondary structure of GlmZ (resp. GlmY). The first six rows show maximum and average results for Sankoff, CalculateScores-1struct and CalculateScores-2structs algorithms. The last two rows represent values obtained for the PAML root ancestral sequence reconstructed on the GlmZ family and on the GlmY family

We observe that, on average, the percentages of canonical basepairs are all the same on the GlmZ structure (99.1 %), but it is 1.5 % higher for the solutions of CalculateScores-2structs on the GlmY structure. Although this is not a huge difference, the fact that we get more canonical basepairs on average by inferring a lot less ancestral sequences is interesting. As for the maximums, in all subsets of solutions we get sequences that have 100 % of the canonical basepairs for both structures. The average (resp. max) H-means for the distinct sets of ancestors produced by Sankoff, CalculateScores-1struct and CalculateScores-2structs are roughly similar, indicating that by cutting the solution space with CalculateScores-1struct and CalculateScores-2structs, we do not lose sequences that have significantly better folding properties in regards to both structures.

We then proceeded to do a comparison of our method with the state-of-the-art maximum likelihood ancestral reconstruction program PAML [34]. For clarity, we remind the reader that PAML considers only one family at a time, and returns one ancestor per node. We generated the ancestors using both families separately, GlmZ and GlmY, and compared the two predicted ancestors percentage of canonical basepairs and H-mean with those obtained with the other methods, as shown in Table 3. The two ancestral sequences produced by PAML have percentages of canonical basepairs (and H-mean of PAML GlmY) that are significantly lower than the best and average values of all the other methods.

#### FinP-traJ clan

We also ran the experiment on the FinP-traJ clan. FinP is an antisense ncRNA that can bind to the 5’ UTR region of the traJ mRNA. The binding of those two RNAs represses the the translation of traJ, which in turn represses F-plasmid transfer [35]. Similarly to the Glm clan, we selected bacterial genomes for which the Rfam alignments were available for both families (54 genomes; see the complete list in Additional file 1 Sec. Biological Data) and we did the same preprocessing to prepare the alignments. The phylogeny for the 54 bacterial strains was also taken from PATRIC [32].

Noticeably, both families in this clan are sequentially and structurally more different than with the Glm clan. The Fitch, Sankoff, and CalculateScores-1struct methods inferred the same ensemble of 12 582 912 sequences at the root. In contrast, CalculateScores- 2structs inferred a strict subset (4x smaller) of 3 145 728 sequences. Running times were of 17 seconds for both Fitch and Sankoff, and 19 seconds for both CalculateScores-1struct and CalculateScores-2structs.

As with Glm, we sampled 200 000 distinct sequences to compare the two sets. We present in the first two rows of Table 4 the maximum and average results for the sampled sequences in Others, the set inferred by Fitch, Sankoff, and CalculateScores-1struct but not by CalculateScores-2structs. The following two rows present those sampled in the subset inferred by CalculateScores-2structs.

**Table 4:** Maximum and average results for the FinP-traJ Clan

	%F	%t	H-mean	EnSF	FreqSF	EnSt	FreqSt
Others	94.7^a^	100^a^	6.25e-01	-23.7	6.25e-01	-23.7	6.25e-01
average	86.8	88.2	3.17e-03	-27.5	1.97e-02	-24.4	3.65e-03
2struct	94.7^a^	100^a^	5.58e-01	-23.7	5.58e-01	-23.7	5.58e-01
average	85.5	91.2	3.86e-03	-27.0	9.62e-03	-25.3	1.39e-02
PAML FinP	100	82.4	7.13e-08	-28.3	2.59e-03	-21.4	3.56e-08
PAML traJ	78.9	100	2.61e-07	-26.3	2.84e-07	-26.2	2.42e-07

The %F (resp. %t) column shows the percentage of all structured positions in the FinP (resp. traJ) family for which the ancestral sequences can form canonical basepairs. The H-mean column represents the harmonic mean. The EnSF column (resp. EnSt) shows the energy of the sequence when folded in the secondary structure of the family FinP (resp. traJ). The FreqSF column (resp. FreqSt) shows the frequency in the ensemble of the secondary structure of FinP (resp. traJ). The first four rows show maximum and average results for the first three algorithms (Others) and CalculateScores-2structs. The last two rows represent values obtained for the PAML root ancestral sequence reconstructed on the FinP family and on the traJ family.

^a^The maximum values showed are the ones maximizing the basepairs in traJ; the ones maximizing FinP are 100 and 94.1 for %F and %t respectively

We show the results with their standard deviation in Additional file 1: Tab. 8.

We observe that on average, the solutions from the “others” group can form 86.8 % of the basepairs of the FinP structure and 88.2 % of the ones of traJ. On the other hand, the subset of ancestors produced by CalculateScores-2structs can form on average 85.5 % (1.3 % less) of the basepairs of the FinP structure and 91.2 % (3 % more) of the ones of traJ, which, overall, seems to be a better compromise. Note that this was achieved by inferring 4 times fewer ancestors.

Regarding the H-mean, the samples taken from the smaller subset of ancestral sequences reconstructed by CalculateScores-2structs show similar results for the maximum and slightly better for the average H-mean than the bigger sets inferred by the other algorithms, which tends to show that our method is not discarding sequences with better folding properties.

We also compared our results with PAML, for each family separately. We observe a stark contrast with our results when comparing the percentage of canonical basepairs for both families. While PAML can get 100 % on the considered structure, it gets only about 80 % of the basepairs of the other structure. When looking at the stability of the functional structures of the two families on the reconstructed ancestral sequences, we observe that our solutions offer a better trade-off (i.e. the average harmonic mean is several degrees of magnitude better that the ones obtained by PAML).

These results suggest that our methods are indeed capable to retrieve ancestral sequences with better fitness to both functional structures of the homologous RNA families. Since RNA families are known to favour the conservation of structures over sequences, we argue that achARNement solutions are better ancestral candidates.

## Conclusions

In this paper, we presented two novel maximum parsimony algorithms, implemented in achARNement, to solve the simultaneous ancestral reconstruction of two ncRNA families sharing a common ancestor. We first evaluated our methods on simulated data, as described in Sec. Simulated data generation, then on two Rfam clans, the Glm and FinP-traJ clan (Sec. Evaluation on biological data).

We first showed that on simulated data, achARNement produces smaller sets of ancestral sequences with fewer errors on average than the classical Fitch and Sankoff algorithms. Since all the ancestral sequences reconstructed at the root by achARNement are included in those produced by the Sankoff algorithm, it indicates that considering the secondary structures does not generate superfluous mutations. Most importantly, considering both structures in CalculateScores-2structs produces orders of magnitudes fewer sequences while always improving on the other algorithms in terms of the average percentage of errors.

The biological data cannot be validated directly, yet some interesting observations can be made. To the best of our knowledge, achARNement has the first implementations of complete parsimonious models able to reconstruct ancestral sequences of large alignments with multiple structures. On both the Glm and FinP-traj clans, CalculateScores-2structs has been shown to infer smaller sets of ancestral sequences than Fitch and Sankoff, while offering a better compromise in terms of the percentage of canonical basepairs for both structures (without penalizing the folding properties, as shown with the similar values of H-mean). Also, the comparison with PAML highlights the benefits of our approach, especially on the FinP-traJ clan, where it is clear that we are able to infer sequences that have better folding properties in both considered structures.

The evolutionary model and algorithms presented here constitute a first attempt at tackling this specific problem. Although the results are encouraging, a lot more work needs to be done in the future to improve our approach: analyzing more in depth the different parameters of our method, reducing even more the number of ancestral sequences inferred and testing on more Rfam clans are just a few examples. The frequency in the ensemble also raises important questions in regards to how we view neutral networks. Given a ncRNA and its functional conformation, what is the minimal frequency in the structures ensemble needed in order for it to be able to fulfill its function? Equally for the RNA design problem, most methods are based on local searches, from a random search as in RNAinverse [28] or with an ant algorithm as in antaRNA-ant [36]. The observed diversity in the quality of sequences at a minimal distance from each other demonstrates the need of more global tools, like the one of IncaRNAtion [37] for example.

Through the annotation of Rfam families, manual curation is needed to distinguish between families of similar sequences with known distinct function or structure, which are joined into clans [26]. In practice, achARNement could be used for the classification of sequences to the correct clan member. achARNement could also be customized to detect families of sequences folding into multiple structures, as those exhibited in [38, 39].

## Additional file

Additional file 1 The file contains the algorithms of CalculateScores-1struct and CalculateScores- 2structs. It is followed by the running times of Fitch, Sankoff, CalculateScores-1struct and CalculateScores- 2structs on the simulated data sets. It also contains the list of bacterial strains used in the biological analysis. We also present additional results on the Glm and FinP-traJ clans reconstructions. (425 KB PDF)
