# Supplementary material for: Reconstruction of ancestral RNA sequences under multiple structural constraints
Source: BMC Genomics. 2016 Nov 11;17(Suppl 10):862. doi: 10.1186/s12864-016-3105-4 (PMC5123390; doi:10.1186/s12864-016-3105-4)
Supplement: Supplementary file 1 — The file contains the algorithms of CalculateScores-1struct and CalculateScores- 2structs. It is followed by the running times of Fitch, Sankoff, CalculateScores-1struct and CalculateScores- 2structs on the simulated data sets. It also contains the list of bacterial strains used in the biological analysis. We also present additional results on the Glm and FinP-traJ clans reconstructions. (425 KB PDF) [file 12864_2016_3105_MOESM1_ESM.pdf]

Supplementary Material

Algorithms

Algorithm 1: `CalculateCosts`(n) This method represents the bottom-up step, where we do a post-order traversal of the tree in order to calculate the costs. Both of the proposed algorithms begin with a call to this method on the root of the species tree, and line 15 shows exactly where the two proposed methods differ.

Data: Given a node n of the species tree with extent sequences at the leaves for the structure families studied

Result: Computes a cost matrix $n.costMatrix$ for every possible nucleotide or di-nucleotide at every internal node of the tree for every structure family

```
1  if n is a leaf then
2      All cells in costMatrix are initialized to ∞;
3      for every structure family fam do
4          for every position pos in the sequence seq do
5              if pos is unpaired in the structure then
6                  n.costMatrix[fam][pos][seq(pos)] = 0;
7              else if pos is paired with posPair in the structure then
8                  dinucleo = seq(pos) + seq(posPair);
9                  n.costMatrix[fam][pos][dinucleo] = 0;
10 else
11     CalculateCosts(n.leftChild);
12     CalculateCosts(n.rightChild);
13     for every structure family fam do
14         for every position 'pos' in the sequence seq do
15             CalculateScores-1struct(n, fam, pos) or
                 CalculateScores-2structs(n, fam, pos);
```

Algorithm 2: `CalculateScores-1struct`(n, fam, pos) The method that corresponds to the first proposed algorithm. It first checks if the position pos is paired or unpaired in the family fam (line 1). If the position is unpaired (represented by -1), a regular Sankoff algorithm (`CalculateScores-Sankoff`, algorithm not shown here) is used to calculate the optimal costs using the substitution matrix only. When the position pos is paired, we calculate the cost of every possible di-nucleotide representing the possible basepairs (16 possible basepairs). More precisely, the substitution cost for each of the nucleotides in the pair (taken from Tab. 1(a)) and the basepair cost (taken from Tab. 1(b)) are considered (see lines 9 and 12). The minimum cost on the left branch is added to the minimum cost on the right branch and constitutes the minimum cost that is stored in the $costMatrix$ attribute (for both the position pos and the paired position $posPair$; see lines 15 and 17).

Data: Given a node n in the species tree, a structure family fam, a position pos
Result: Compute and update $n.costMatrix[fam][pos]$ using the information of one structure family

```
1  posPair = position paired with pos in the family fam, or −1 if unpaired;
2  if posPair == -1 then
3  |   CalculateScores-Sankoff(n, fam, pos);
4  else if pos < posPair then
5  |   for every possible di-nucleotide dinuc do
6  |   |   minLeft = ∞;
7  |   |   minRight = ∞;
8  |   |   for every possible di-nucleotide dinucChild do
9  |   |   |   left = n.leftChild.costMatrix[fam][pos][dinucChild] +
   |   |   |     substitutionCost(dinucChild, dinuc) + basePairCost(dinuc);
10 |   |   |   if left < minLeft then
11 |   |   |   |   minLeft = left;
12 |   |   |   right = n.rightChild.costMatrix[fam][pos][dinucChild] +
   |   |   |     substitutionCost(dinucChild, dinuc) + basePairCost(dinuc);
13 |   |   |   if right < minRight then
14 |   |   |   |   minRight = right;
15 |   |   n.costMatrix[fam][pos][dinuc] = minLeft + minRight;
16 |   |   revDinuc = dinuc[1] + dinuc[0];
17 |   |   n.costMatrix[fam][posPair][revDinuc] = minLeft + minRight;
```

Algorithm 3: `CalculateScores-2structs`(n, fam, pos) The method that corresponds to the second proposed algorithm, which considers both structures. The simpler case is when the position pos is unpaired(see lines 2 to 13). Then, only the position paired with pos in the other structure needs to be considered, if it is paired (see Algorithm 4. The more complex case is when position pos is paired. (see lines 14 to 27). We then use Algorithm 5 to evaluate the costs. Basically, it is very similar to Algorithm 4, except that in this case, we also have to check for the position paired with $posPair$ in the other structure.

Data: Given a node n in the species tree, a structure family fam, a position pos
Result: Compute and update $n.costMatrix[fam][pos]$ using the information of both structure families

1 $posPair$ = position paired with pos in the family fam, or -1 if unpaired;
2 **if** $posPair$ == -1 **then**
3   **for** every possible nucleotide nuc **do**
4         $minLeft = \infty$;
5         $minRight = \infty$;
6   **for** every possible nucleotide $nucChild$ **do**
7             $left$ = `MutCost-unpaired`$(n.leftChild, fam, pos, nuc, nucChild)$;
8   **if** $left < minLeft$ **then**
9                 $minLeft = left$;
10             $right$ = `MutCost-unpaired`$(n.rightChild, fam, pos, nuc, nucChild)$;
11   **if** $right < minRight$ **then**
12                 $minRight = right$;
13         $n.costMatrix[fam][pos][nuc] = minLeft + minRight$;
14 **else if** $pos < posPair$ **then**
15   **for** every possible di-nucleotide $dinuc$ **do**
16         $minLeft = \infty$;
17         $minRight = \infty$;
18   **for** every possible di-nucleotide $dinucChild$ **do**
19             $left$ = `MutCost-paired`$(n.leftChild, fam, pos, dinuc, dinucChild)$;
20   **if** $left < minLeft$ **then**
21             $minLeft = left$;
22         $right$ = `MutCost-paired`$(n.rightChild, fam, pos, dinuc, dinucChild)$;
23   **if** $right < minRight$ **then**
24         $minRight = right$;
25     $n.costMatrix[fam][pos][dinuc] = minLeft + minRight$;
26     $revDinuc = dinuc[1] + dinuc[0]$;
27     $n.costMatrix[fam][posPair][revDinuc] = minLeft + minRight$;

Algorithm 4: `MutCost-unpaired`$(n, fam, pos, nuc, nucChild)$ Called when there is no basepair in the current structure, this method verifies the position paired with pos in the other structure to calculate the cost. Since the position paired with pos ($otherPosPair$) is not fixed during this step, we calculate an average cost over all possible nucleotides at that other position (see line 5).

Data: Given a node n in the species tree, a structure family fam, a position pos, and two nucleotides nuc and $nucChild$
Result: Return the cost of mutating nucChild to nuc

1 $costThisStruct = n.costMatrix[fam][pos][nucChild] + substitutionCost(nucChild, nuc)$;
2 $costOtherStruct = 0$;
3 $otherPosPair$ = position paired with pos in the other family;
4 **if** $otherPosPair$!= -1 **then**
5     $costOtherStruct = \sum_{nc=A,C,G,U} basePairCost(nuc + nc)/4$;
6 **return** $costThisStruct + (1 - G) * costOtherStruct$;

Algorithm 5: `MutCost-paired`$(n, fam, pos, dinuc, dinucChild)$ Called when there is a basepair in the current structure, this method verifies the positions paired with pos and $posPair$ in the other structure to calculate the cost. If the basepairs are the same in both structures, then we simply return 100% of the value of basePairCost(dinuc) (see line 6). Otherwise, since the positions paired with pos and $posPair$ are not fixed during this step, we calculate an average cost over all possible nucleotides at that other position (see lines 7 and 10).

Data: Given a node n in the species tree, a structure family fam, a position pos, and two pairs of nucleotides $dinuc$ and $dinucChild$
Result: Return the cost of mutating $dinucChild$ to $dinuc$

1 $costThisStruct = n.costMatrix[fam][pos][dinucChild] + substitutionCost(dinucChild, dinuc) + G * basePairCost(dinuc)$;
2 $costOtherStruct = 0$;
3 $otherPosPair$ = position paired with pos in the other family;
4 **if** $otherPosPair$!= -1 **then**
5   **if** $otherPosPair == posPair$ **then**
6   **return** $costThisStruct + (1 - G) * basePairCost(dinuc)$;
7     $costOtherStruct += \sum_{nc=A,C,G,U} basePairCost(dinuc[0] + nc)/4$;
8 $otherPosPair$ = position paired with $posPair$ in the other family;
9 **if** $otherPosPair$!= -1 **then**
10     $costOtherStruct += \sum_{nc=A,C,G,U} basePairCost(dinuc[1] + nc)/4$;
11 **return** $costThisStruct + (1 - G) * costOtherStruct$;

Algorithm 6: `FindOptimalSequences`(n) Contains the middle and top-down steps of the algorithms. Line 4 shows the middle step, which is when we do a regular Fitch on the cost matrices of both families to get the cost matrix for the final ancestor (before the duplication). The rest of the method shows how we enumerate the optimal sequences based on the cost matrices.

Data: Given a node n of the species tree with costMatrix calculated for every node
Result: Computes a list of optimal sequences $listOptSeqs$ for every structure family at every internal node of the tree

1 **if** n is a leaf **then**
2   **return**
3 **else if** n is the root **then**
4     $n.costMatrix$ = `CalculateScores-Fitch`$(n.costMatrix[fam0], n.costMatrix[fam1])$;
5   **for** every position pos **do**
6         $listOptNucleos[pos]$ = the list of nucleotides of minimum cost from $n.costMatrix[pos]$ for that position;
7     $n.listOptSeqs[fam0] = n.listOptSeqs[fam1]$ = enumerate all possible sequences using $listOptNucleos$;
8 **else**
9   **for** every structure family fam **do**
10   **for** every $parentSeq$ in $n.parent.listOptSeqs[fam]$ **do**
11   **for** every position pos **do**
12   Update $listOptNucleos[pos]$ using `CalculateScores-1struct`(n, fam, pos) or `CalculateScores-2structs`(n, fam, pos);
13         $n.listOptSeqs[fam]$ = enumerate all possible sequences using $listOptNucleos$;
14 `FindOptimalSequences`$(n.leftChild)$;
15 `FindOptimalSequences`$(n.rightChild)$;

Running times

Here we show the running times of the four algorithms tested on the simulated data sets. Fig. 1 shows the average running times for the three different pairs of structures when inferring the ancestral sequences at all the ancestral nodes of the species tree. Interestingly, we can see that the average running times of the Fitch and Sankoff algorithms are much higher than the ones of `CalculateScores-1struct` and `CalculateScores-2structs` for the pair of structures 01, which can be explained by the fact that Fitch and Sankoff were inferring a much larger number of optimal ancestral sequences. Even though `CalculateScores-1struct` and `CalculateScores-2structs` are a little bit slower in general (because of the constant number of additional calculations required), Fitch and Sankoff algorithms were getting slowed down by the larger number of optimal solutions.

`achARNement` also offers the possibility to infer the ancestral sequences at the root of the tree only, which is convenient when we are mostly interested by the original ancestral sequences. This option was useful for producing the results on the biological data sets. Fig. 2 shows the average running times when inferring the ancestral sequences at the root only. In this figure, we can clearly see the difference in running time caused by the additional calculations required by `CalculateScores-1struct` and `CalculateScores-2structs` to calculate the minimal costs during the bottom-up step.

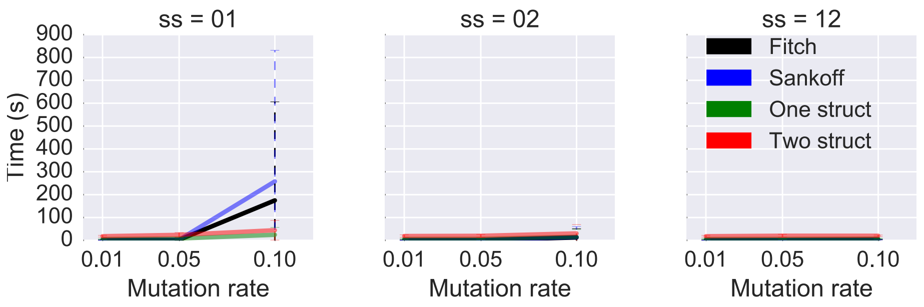

Figure 1: Average running time (in seconds), on the simulated data sets, of Fitch, Sankoff, `CalculateScores-1struct` and `CalculateScores-2structs` for the inference of the ancestral sequences in the entire tree. Each column represents a different pair of secondary structures. For three mutation rates: 1%, 5% and 10%.

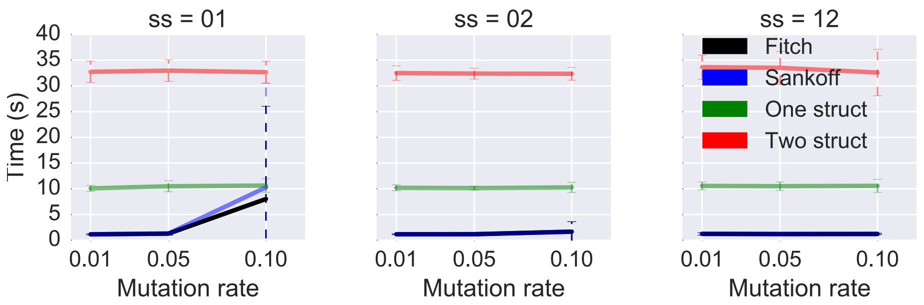

Figure 2: Average running time (in seconds), on the simulated data sets, of Fitch, Sankoff, `CalculateScores-1struct` and `CalculateScores-2structs` for the inference of the ancestral sequences at the root only. Each column represents a different pair of secondary structures. For three mutation rates: 1%, 5% and 10%.

Biological Data

Table 4: All strains used for the Glm clan analyses (Sec. Evaluation on biological data - Glm clan) with their Rfam accession number (Part 1/2)

Strain	Rfam sequence identifier
Escherichia_coli_KO11	CP002516.1
Escherichia_coli_NA114	CP002797.2
Cronobacter_sakazakii_ATCC_BAA-894	CP000783.1
Escherichia_coli_55989	CU928145.2
Escherichia_coli_UM146	CP002167.1
Salmonella_enterica_subsp._enterica_serovar_Typhimurium_str._UK-1	CP002614.1
Enterobacter_cloacae_subsp._cloacae_ATCC_13047	CP001918.1
Escherichia_coli_ETEC_H10407	FN649414.1
Edwardsiella_tarda_EIB202	CP001135.1
Escherichia_coli_ATCC_8739	CP000946.1
Klebsiella_pneumoniae_subsp._pneumoniae_NTUH-K2044	AP006725.1
Escherichia_coli_E24377A	CP000800.1
Salmonella_enterica_subsp._enterica_serovar_Schwarzengrund_str._SL480	ABEJ01000018.1
Escherichia_coli_SE11	AP009240.1
Salmonella_enterica_subsp._enterica_serovar_Enteritidis_str._P125109	AM933172.1
Escherichia_coli_536	CP000247.1
Escherichia_coli_IAI39	CU928164.2
Edwardsiella_tarda_FL6-60	CP002154.1
Escherichia_coli_APEC_O1	CP000468.1
Shigella_sonnei_Ss046	CP000038.1
Proteus_penneri_ATCC_35198	ABVP01000019.1
Shigella_flexneri_2002017	CP001383.1
Escherichia_coli_BW2952	CP001396.1
Escherichia_coli_IHE3034	CP001969.1
Salmonella_enterica_subsp._enterica_serovar_Choleraesuis_str._SC-B67	AE017220.1
Citrobacter_koseri_ATCC_BAA-895	CP000822.1
Escherichia_coli_SMS-3-5	CP000970.1
Dickeya_dadantii_3937	CP002038.1
Shigella_boydii_Sb227	CP000036.1
Escherichia_coli_str._K-12_substr._MG1655	U00096.2
Shigella_boydii_CDC_3083-94	CP001063.1
Escherichia_coli_SE15	AP009378.1
Escherichia_coli_DH1	CP001637.1
Shigella_flexneri_2a_str._301	AE005674.2
Cronobacter_turicensis_z3032	FN543093.2
Pantoea_ananatis_PA13	CP003085.1

Table 5: All strains used for the Glm clan analyses (Sec. Evaluation on biological data - Glm clan) with their `Rfam` accession number (Part 2/2)

**Strain**	`Rfam` **sequence identifier**
Escherichia_coli_ED1a	CU928162.2
Yersinia_pseudotuberculosis_IP_31758	CP000720.1
Klebsiella_pneumoniae_342	CP000964.1
Citrobacter_freundii_str._ballerup_7851/39	CACD01000298.1
Klebsiella_variicola_At-22	CP001891.1
Providencia_rustigianii_DSM_4541	ABXV02000023.1
Escherichia_coli_LF82	CU651637.1
Escherichia_coli_UMNK88	CP002729.1
Escherichia_coli_W	CP002185.1
Salmonella_enterica_subsp._enterica_serovar_Newport_str._SL254	CP001113.1
Yersinia_pestis_biovar_Orientalis_str._IP275	AAOS02000021.1
Yersinia_pestis_biovar_Orientalis_str._India_195	ACNR01000027.1
Salmonella_enterica_subsp._enterica_serovar_Paratyphi_A_str._AKU_12601	FM200053.1
Escherichia_coli_HS	CP000802.1
Pectobacterium_wasabiae_WPP163	CP001790.1
Erwinia_sp._Ejp617	CP002124.1
Yersinia_enterocolitica_subsp._enterocolitica_8081	AM286415.1
Shigella_flexneri_5_str._8401	CP000266.1
Escherichia_coli_ABU_83972	CP001671.1
Escherichia_coli_UTI89	CP000243.1
Salmonella_enterica_subsp._enterica_serovar_Dublin_str._CT_02021853	CP001144.1
Escherichia_coli_CFT073	AE014075.1
Escherichia_coli_042	FN554766.1
Yersinia_pestis_Antiqua	CP000308.1
Serratia_sp._AS13	CP002775.1
Klebsiella_pneumoniae_subsp._pneumoniae_MGH_78578	CP000647.1
Yersinia_pestis_CO92	AL590842.1
Escherichia_coli_IAI1	CU928160.2
Escherichia_coli_UMN026	CU928163.2
Escherichia_coli_S88	CU928161.2
Providencia_stuartii_ATCC_25827	ABJD02000102.1
Salmonella_enterica_subsp._enterica_serovar_Montevideo_str._2009085258	AETP01000030.1
Salmonella_enterica_subsp._enterica_serovar_Typhi_str._E98-3139	CAAZ01000080.1
Enterobacter_asburiae_LF7a	CP003026.1
Serratia_plymuthica_AS9	CP002773.1
Escherichia_coli_B_str._REL606	CP000819.1
Salmonella_enterica_subsp._enterica_serovar_Heidelberg_str._SL486	ABEL01000007.1
Yersinia_pestis_biovar_Medievalis_str._Harbin_35	CP001608.1

Table 6: All strains used for the FinP-traJ clan analyses (Sec. Evaluation on biological data - FinP-Traj) with their Rfam accession number.

Strain	Rfam sequence identifier
Escherichia coli M718	ADAW01000063.1
Escherichia coli 3.2303	AFAE01000017.1
Escherichia coli 2.3916	AFAB01000162.1
Escherichia coli UMNF18	AGTD01000003.1
Escherichia coli H736	ADAU01000091.1
Escherichia coli B41	AFAH01000026.1
Escherichia coli ETEC H10407	AP010910.1
Escherichia coli UMNK88	CP002732.1
Escherichia coli H489	AEHX01000116.1
Escherichia coli 101-1	AAMK02000010.1
Escherichia coli 53638	CP001065.1
Shigella dysenteriae 1012	AAMJ02000017.1
Escherichia coli 99.0741	AEZR01000016.1
Escherichia coli E1167	AEHS01000059.1
Escherichia coli 5.0588	AEZK01000005.1
Escherichia coli B088	ACXE01000024.1
Escherichia coli H120	AEHV01000116.1
Escherichia coli 1.2264	AEZO01000045.1
Escherichia coli 97.0264	AEZP01000093.1
Escherichia coli TA271	ADAZ01000085.1
Escherichia coli H591	ADBB01000009.1
Escherichia coli B7A	AAJT02000107.1
Escherichia coli 9.0111	AEZZ01000025.1
Escherichia coli E22	AAJV02000038.1
Escherichia coli 3.2608	AEZS01000057.1
Escherichia coli 93.0624	AEZT01000020.1
Escherichia coli 4.0967	AFAA01000070.1
Escherichia coli E110019	AAJW02000025.1
Escherichia coli 4.0522	AEZU01000067.1
Escherichia coli 97.0246	AEZJ01000074.1
Escherichia coli 97.0259	AEZL01000004.1
Escherichia coli PCN033	AFAT01000151.1
Escherichia coli 042	FN554767.1
Escherichia coli FVEC1302	ACXH01000019.1
Escherichia coli FVEC1412	ACXI01000054.1
Escherichia coli H299	ADBC01000169.1
Escherichia coli HM605	CADZ01000093.1
Escherichia coli APEC O1	DQ381420.1
Escherichia coli H252	AEFI01000032.1
Escherichia coli H263	AEFJ01000092.1
Escherichia sp. 3 2 53FAA	ACAC01000148.1
Escherichia coli UM146	CP002168.1
Escherichia coli UTI89	CP000244.1
Escherichia coli F11	AAJU02000030.1
Escherichia coli M605	ADAV01000058.1
Escherichia coli SE15	AP009379.1
Escherichia coli SMS-3-5	CP000971.1
Escherichia coli TW10509	AEHW01000038.1
Escherichia sp. TW10509	AEKA01000361.1
Escherichia coli M863	AEHZ01000077.1
Escherichia coli 1.2741	AEZI01000031.1
Escherichia fergusonii ECD227	AEVY01000077.1
Salmonella enterica subsp. enterica serovar Typhimurium str. LT2	AE006471.1
Klebsiella pneumoniae 1191100241	AFXH01000092.1
Klebsiella pneumoniae subsp. pneumoniae MGH 78578	CP000648.1

GLM and FinP-Traj Results

	%Z (Std. Dev.)	%Y (Std. Dev.)	H-mean (Std. Dev.)	
Sankoff	99.1 (1.54)	93.0 (3.41)	6.61e-06 (3.25e-05)	
1struct	99.1 (1.54)	93.0 (3.41)	9.02e-05 (3.04e-04)	
2struct	99.1 (1.54)	94.5 (3.03)	7.45e-06 (4.45e-05)	
	EnSZ (Std. Dev.)	FreqSZ (Std. Dev.)	EnSY (Std. Dev.)	FreqSY (Std. Dev.)
Sankoff	-15.5 (1.79)	4.09e-06 (2.39e-05)	-17.8 (2.05)	1.55e-04 (5.65e-04)
1struct	-16.1 (1.79)	5.54e-05 (2.13e-04)	-17.4 (1.78)	5.68e-04 (1.54e-03)
2struct	-16.1 (1.79)	3.78e-06 (2.29e-05)	-19.4 (1.78)	9.27e-04 (2.63e-03)

Table 7: Average results for the Glm Clan, with standard deviations, for `Sankoff`, `CalculateScores-1struct` and `CalculateScores-2structs` algorithms. The %Z (resp. %Y) column shows the percentage of all structured positions in the GlmZ (resp. GlmY) family for which the ancestral sequences can form canonical basepairs. The H-mean column represents the harmonic mean. The EnSZ column (resp. EnSY) shows the energy of the sequence when folded in the secondary structure of the family GlmZ (resp. GlmY). The FreqSZ column (resp. FreqSY) shows the frequency in the ensemble of the secondary structure of GlmZ (resp. GlmY).

	%F (Std. Dev)	%t (Std. Dev.)	H-mean (Std. Dev.)	
Others	86.8 (5.58)	88.2 (5.88)	3.17e-03 (2.35e-02)	
2struct	85.5 (5.74)	91.2 (5.09)	3.86e-03 (2.26e-02)	
	EnSF (Std. Dev.)	FreqSF (Std. Dev.)	EnSt (Std. Dev.)	FreqSt (Std. Dev.)
Others	-27.5 (1.95)	1.97e-02 (6.39e-02)	-24.4 (1.41)	3.65e-03 (2.57e-02)
2struct	-27.0 (3.27)	9.62e-03 (4.07e-02)	-25.3 (2.02)	1.39e-02 (5.12e-02)

Table 8: Average results for the FinP-traJ Clan, with standard deviations, for the first three algorithms (`Others` and `CalculateScores-2structs`. The %F (resp. %t) column shows the percentage of all structured positions in the FinP (resp. traJ) family for which the ancestral sequences can form canonical basepairs. The H-mean column represents the harmonic mean. The EnSF column (resp. EnSt) shows the energy of the sequence when folded in the secondary structure of the family FinP (resp. traJ). The FreqSF column (resp. FreqSt) shows the frequency in the ensemble of the secondary structure of FinP (resp. traj).
